# In Vitro and In Silico Characterization of Curcumin-Loaded Chitosan–PVA Hydrogels: Antimicrobial and Potential Wound Healing Activity

**DOI:** 10.3390/gels9050394

**Published:** 2023-05-09

**Authors:** Hitesh Chopra, Shabana Bibi, Yugal Kishore Mohanta, Tapan Kumar Mohanta, Sandeep Kumar, Inderbir Singh, Muhammad Saad Khan, Pradipta Ranjan Rauta, Abdulrahman Alshammari, Metab Alharbi, Abdullah F. Alasmari

**Affiliations:** 1 Chitkara College of Pharmacy, Chitkara University, Rajpura 140401, Punjab, India; chopraontheride@gmail.com; 2 Department of Biosciences, Shifa Tameer-e-Millat University, Islamabad 44000, Pakistan; 3 Yunnan Herbal Laboratory, College of Ecology and Environmental Sciences, Yunnan University, Kunming 650091, China; 4 Nano-Biotechnology and Translational Knowledge Laboratory, Department of Applied Biology, School of Biological Sciences, University of Science and Technology, Meghalaya (USTM), Techno City, 9th Mile, Baridua, Ri-Bhoi 793101, Meghalaya, India; ykmohanta@gmail.com; 5 Natural and Medical Sciences Research Centre, University of Nizwa, Nizwa 616, Oman; nostoc.tapan@gmail.com; 6 Amar Shaheed Baba Ajit Singh Jujhar Singh Memorial College of Pharmacy, Bela, Ropar 140111, Punjab, India; sandeep_pharm70@yahoo.com; 7 Department of Biosciences, Faculty of Sciences, COMSATS University Islamabad, Sahiwal 57000, Pakistan; saad.khan@cuisahiwal.edu.pk; 8 Department of Biological Sciences, AIPH University, EAST Campus, Prachi Vihar Anantapur, Phulnakhara, Bhubaneswar 754001, Odisha, India; prrauta@aiph.ac.in; 9 Department of Pharmacology and Toxicology, College of Pharmacy, King Saud University, P.O. Box 2455, Riyadh 11451, Saudi Arabia; abdalshammari@ksu.edu.sa (A.A.); mesalharbi@ksu.edu.sa (M.A.); afalasmari@ksu.edu.sa (A.F.A.)

**Keywords:** curcumin, hydrogels, natural products, molecular characterization, antimicrobial potential

## Abstract

Curcumin has been used in traditional medicine forages. The present study aimed to develop a curcumin-based hydrogel system and assess its antimicrobial potential and wound healing (WH) activity on an invitro and in silico basis. A topical hydrogel was prepared using chitosan, PVA, and Curcumin in varied ratios, and hydrogels were evaluated for physicochemical properties. The hydrogel showed antimicrobial activity against both gram-positive and gram-negative microorganisms. In silico studies showed good binding energy scores and significant interaction of curcumin components with key residues of inflammatory proteins that help in WH activity. Dissolution studies showed sustained release of curcumin. Overall, the results indicated wound healing potential of chitosan–PVA–curcumin hydrogel films. Further in vivo experiments are needed to evaluate the clinical efficacy of such films for wound healing.

## 1. Introduction

The skin is an essential organ of the body’s defense system, defending against germs, ultraviolet radiation, and injuries [1]. It controls body temperature, and enables absorption of vitamin D through sunlight [2]. While apparent solitary wounds affect the epidermis, fractional thickness and full thickness wounds cause harm to the dermis, subcutaneous, fatty tissues, and/or bone [3,4,5,6]. The reconnecting of skin edges using sutures and surgical adhesives is the primary objective of wound healing (WH). On the other hand, the secondary purpose describes how a wound heals when the skin borders are not forced together and the wound must close and fill with granulating tissues [7].Wound can be defined as a tear in the skin or other bodily tissues resulting from an accident or surgical incision [8,9,10]. These may be caused by a variety of forms of trauma, and it is crucial that wounds be cleansed and dressed properly in order to prevent the spread of infection and additional harm [11]. Wound care is the ongoing management of a wound by preserving conditions that are favorable to healing, both directly and indirectly, and by stopping skin deterioration [12]. The optimal management of a wound depends on its size, depth, severity, and location throughout the care period. Due to technical developments that are revealing more information on the genesis and healing of wounds, this treatment is advancing quickly. Hydrogels are one of several solutions for the WH process that are available based on the severity of the lesion [13]. Hydrogels are a realistic three-dimensional linkage of hydrophilic polymers that fasten together and swell in water. When applied as a wound dressing, hydrogels not only produce a physical blockade and absorb extra exudate; they offers a humid environment that promotes WH [14,15,16]. In addition, hydrogels can properly fill irregularly shaped wounds and effectively control heavy bleeding. It is possible to modify hydrogel qualities such as composition, sensitivity to wound stimuli, and more, depending on the intended use. Antiseptic agents, antibiotics, anti-inflammatory, and antioxidants may all be delivered to the wound to combat infection and chronic wound problems. The healing prospects of skin and related stem cells may be supported and maximized by using hydrogels to transport bioactive compounds known to speed up WH and to enhance angiogenesis, re-epithelialization, and new extracellular matrix (ECM) synthesis and maturation. While many natural products have been reported to possess WH properties, they typically require delivery systems that can deliver them to the target site.

**Figure 1:**
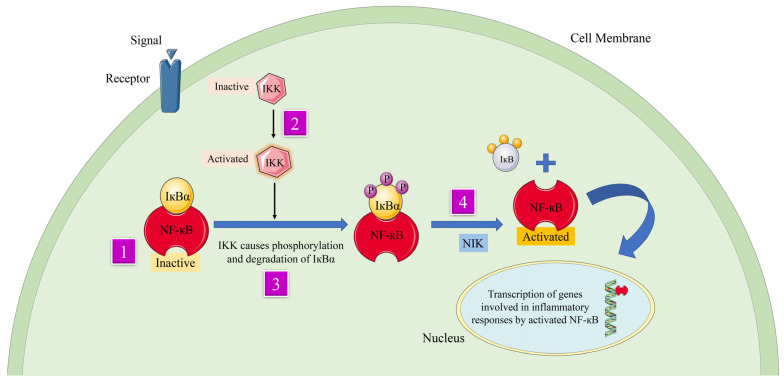
Curcumindeals with the mechanism of inflammation and signaling pathways by suppressing transcription factor NFκB. The numbers 1, 2, and 3 in the figure indicate different pathways involved in the activity of the bioactive compoundthat makes up Curcumin. Parts of the figure were drawn using pictures from Servier Medical Art. Servier Medical Art by Servier is licensed under a Creative Commons Attribution 3.0 Unported License (https://creativecommons.org/licenses/by/3.0/) (assessed on 3 November 2022).

Turmeric, a herb in the ginger family, has long been used in Indian and Chinese cooking as a culinary spice and as a food dye [17]. The plant’s root, or rhizome, has long been utilized in traditional Indian and Chinese medicine, and is the most medicinally beneficial element of the whole plant. Curcumin is a staple in Indian traditional medicine, and is used in the treatment of biliary problems, cough, diabetic ulcers, hepatic disorders, rheumatism, and sinusitis. In turmeric, curcumin has three derivatives as curcuminoids, accounting for between 2% and 5% of the spice and 77% of the extract [18]. According to Joe et al., curcumin controls inflammation in multiple ways [19] Tumor necrosis factor alpha (TNF-α) and interleukin-1 (IL-1) are two key cytokines generated by monocytes and macrophages, and are critical regulators of inflammatory responses. Curcumin has been shown to suppress their production. Additionally, curcumin inhibits the activity of NF-(κ)B, a transcription factor known to play a role in the start of inflammatory reactions, by inhibiting the activity of NF-kappa-B (NF-(κ)B), a light chain enhancer of activated B cells. There are a number of kinases (AKT, PI3K, IKK) that ordinarily activate NF-(κ)B, and curcumin impacts a number of these pathways (Figure 1).

According to Frey and Malik, NF-(κ)B was previously determined to be oxidant-sensitive, underlining the mechanism of oxidation and inflammation pathways associated with WH [20]. Curcumin with collagen matrix (CICM) was examined in vitro utilizing the lipid peroxidation technique, revealing curcumin as a potent antioxidant agent against peroxy-radicals. Excision wounds on rats were administered curcumintransdermally, successfully reducing H_2_O_2_-induced damage to keratinocytes and fibroblasts [21]. Curcumin’s ability to protect keratinocytes and fibroblasts against hydrogen peroxide has been shown in vitro [22]. Curcumin is poorly absorbed after oral administration due to its hydrophobicity, and only small amounts of the chemical are seen in the blood serum. Curcumin is likewise a light-sensitive chemical, and undergoes significant first-pass metabolism [23,24]. Curcumin is a good choice for topical usage because of its limited water solubility and significant first-pass metabolism. Curcumin has been shown to have a better impact on WH when applied topically rather than taken orally, which is because of the increased accessibility of the substance at the wound site [25,26]. 

A cationic polysaccharide, chitosan is the partly to completely deacetylated version of the natural cationic polysaccharide chitin [27]. Sources include crustacean shells, the cell walls of fungi and algae, insect exoskeletons, and molluscradula. Unlike chitin, because chitosan is acetylated it can be dissolved in acidic aqueous solutions. Chitosan’s acetylation degree can be described in two ways: first, as a percentage of acetylation (DA percent), and second, as a molar fraction of N-acetylated units (NAF). Chitosan’s antibacterial action is dependent on the polymer chain’s amino groups. Protonation of these amino groups makes chitosan positively charged [28]. Chitosan’s –NH_2_ groups are changed to the soluble protonated NH_3_^+^ form when the pH of the solution falls below its pKa value of 6.3, making it soluble in acidic aqueous solutions [29,30]. Gram-positive bacteria possess a layer of peptidoglycan that contains teichoic acids, which provide the bacterial surface with a negative charge. Gram-negative bacteria have a thick lipopolysaccharide layer that contains lipopolysaccharides. On the other hand, negatively charged molecules are present in the outer membranes of fungal species and outer envelope of viruses [31].

Alcoholysis, hydrolysis, or ammonolysis of poly(vinyl acetate) yields PVA, a water-soluble long-chain polymer (PVAc). Using crosslinking and swelling, PVA-based hydrogels create colloidal dispersions with three-dimensional network architectures. In addition to their excellent mechanical qualities (i.e., high elastic modulus and strength), they exhibit minimal toxicity and high biocompatibility [32,33]. In hydrogels, a solution of high-alcoholysis PVA (over 98 percent) may self-gelate between molecular chains and form hydrogels at room temperature. However, because to the low gelation density of this hydrogel, it has poor mechanical characteristics that limit its use. Different crosslinking techniques and preparation conditions are needed to create an optimal polymer network structure for PVA hydrogels with high mechanical strength, enhanced water concentration, and transparency.

Hence, in this study we formulated curcumin-based antibacterial WH hydrogels. The hydrogels were found to possess WH action as confirmed by insilico studies. An integrated experimental and in silico study was conducted to design and develop novel hydrogels using biodegradable polymers such as chitosan and polyvinyl alcohol (PVA) for the targeted and sustained release of curcumin at the wound site. The main novelty involved in the present research involves green synthesis of hydrogels via physical crosslinking of chitosan and PVA to effectively encapsulate curcumin without any use of chemicals. 

## 2. Results and Discussion

### 2.1. Thickness, Weight Variation, and Folding Endurance

**Figure 2:**
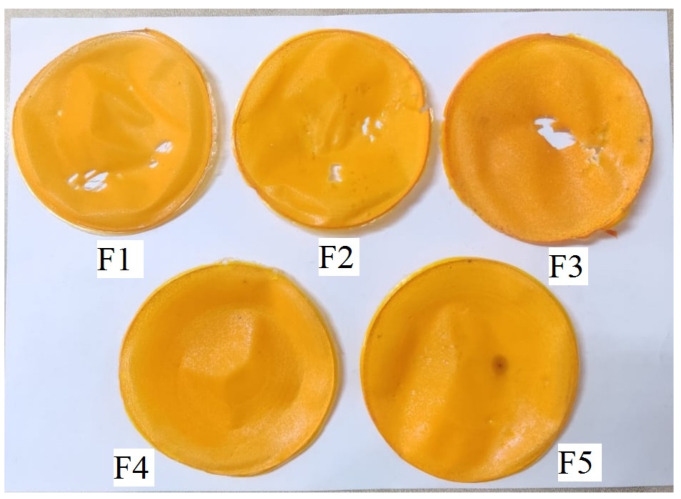
Image showing final prepared films from batches F1–F5.

The prepared films were evaluated based on various physicochemical parametric tests, as shown in Table 1. The physical tests indicate the degree of cross-linking occurring between the various polymers of hydrogel films. The thickness was found to vary from 0.039 ± 0.004 to 0.057 ± 0.006 mm, while the weight of dried films varied from 0.417 ± 0.03 and 0.495 ± 0.02 g. As the concentration of chitosan increased from batch F1 to batch F5, the folding endurance was found to increase from 345 ± 12 to 478 ± 14. The results are tabulated in Table 1, and the prepared films are shown in Figure 2.

### 2.2. Moisture Content and Moisture Uptake

The moisture content percentage of the films was found to increase from 17.14 ± 1.12 to 26.72 ± 2.17% between batches F1 and F5. Cazón et al. have previously reported that higher moisture content improves the water vapor permeability and UV barrier action of films [34]. Chitosan has a large amount of hydrophilic amino and hydroxyl groups, which are responsible for the water absorption capacity of films. Therefore, as the concentration of chitosan in the produced films increased, the moisture content increased proportionately. In addition, the moisture contents of films increased in similar fashion, from 10.28 ± 0.04 to 15.47 ± 0.08%. Moisture content in itself is an important parameter for films, as it is related to the exudate soaking capacity of hydrogels.

### 2.3. Swelling Ratio

**Figure 3:**
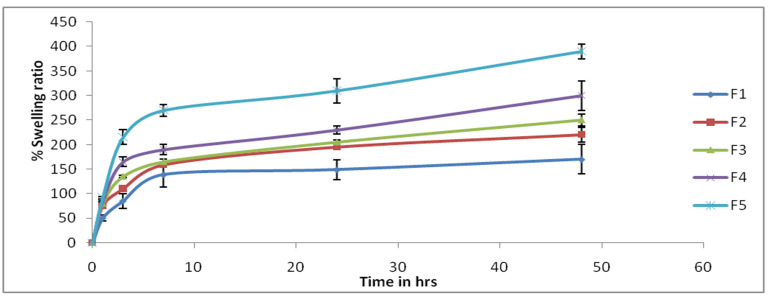
Swelling ratio of hydrogel film batches (n = 3).

The swelling ratio accounts for the fluid uptake capacity of hydrogel films, which can be an important element of the WH action of films. It was found that the swelling ration decreased as the concentration of PVA decreased, and vice versa. Thus, it can be said that the swelling ratio increases with increasing concentration of chitosan (see Figure 3). This may be due to an increase in the cross-linking density of polymeric chains due to increased chitosan content. Similar results for hydrogel swelling behavior have been reported by Abdeen [35] and Casey [36]. 

### 2.4. WVTR

The water vapor transfer rate (WVTR), which directly governs the moisture microenvironment in WH, is a fundamental physical feature of wound dressings that has not yet been explored extensively. WVTR is a useful tool for evaluating a dressing’s efficacy in preventing moisture loss. In this way, a range of wound dressings with varying WVTRs may be used to control the amount of moisture on the wound’s surface. When the WVTR is too high, the wound may dry up, whereas when it is too low the wound can fill up with exudate. To provide the best circumstances for natural healing, a dressing with the right WVTR is necessary. The WVTR is the constant rate at which water vapor diffuses across a film under a given set of parameters for temperature and humidity. During our analysis, it was found that as the chitosan concentration increased from batch F1 to batch F5, the WVTR decreased significantly. The WVTR was found to have a value between 1630.70 ± 25.68 and 2710.54 ± 15.96 g/m^2^/day (Table 2). Similar results for WVTR have previously been reported by Kanatt et al. for chitosan/PVA-based films [37]. Another group of researchers reported similar results in Cassava starch/chitosan-based films, with an increase in chitosan concentration resulting in a decrease in WVTR due to hydrogen bonding between the amino and hydroxyl groups of Cassava starch, causing reduced availability of hydrophilic groups [38]. Typically, a WVTR of 2030 g/m^2^/day is ideal for wound dressing [39].

### 2.5. Tensile Strength and Elongation at Break

The mechanical strength of hydrogel films can be evaluated using the tensile strength and elongation at break. As the concentration of chitosan increased, the tensile strength of films was found to increase from 4.14 ± 0.24 to 38.87 ± 5.24 N, while the elongation at break varied from30.14 ± 1.49 to 35.78 ± 2.58 mm. This may be due to increased cross-linking density between chitosan and PVA.

### 2.6. FTIR (Fourier Transform Infrared) Spectroscopy

**Figure 4:**
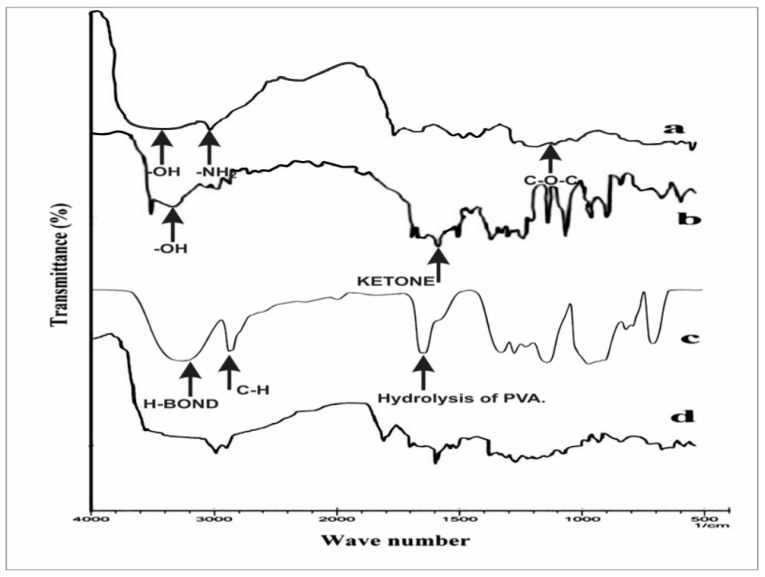
FTIR Spectra for (**a**) chitosan, (**b**) curcumin, (**c**) PVA, and (**d**) hydrogel film.

In Figure 4a, chitosan shows peaks due to −OH and −NH2 stretching near 3500–3000 cm^−1^, a−CH stretching peak at 2850 cm^−1^, and a peak at 1250 cm^−1^ due to C–O–C stretching. Curcumin shows absorption bands near 3500–3000 cm^−1^ due to the presence of phenolic −OH, while the peak at 1600 cm^−1^ is due to the stretching of conjugated ketone (Figure 4b). In the case of PVA, the peak at 2900 cm^−1^ is due to C–H alkyl stretching, the band detected at 3200 cm^−1^ can be attributed to hydrogen bonding (Figure 4c), and the peak at 1700 cm^−1^ is due to hydrolysis of PVA. In the FTIR results for the hydrogel films (Figure 4d), cross-linking occurs between hydrogen and amino groups of compounds. On studying the FTIR graph of the films, it was found that although the wave number underwent mild shifts, the intensity was attenuated at a certain wave number. This may be due to the presence of new bonds formed between interacting compounds. Previously, Yang et al. used FTIR to study the interactions between chitosan and PVA, and reported similar observations [40]. 

### 2.7. SEM (Scanning Electron Microscopy)

**Figure 5:**
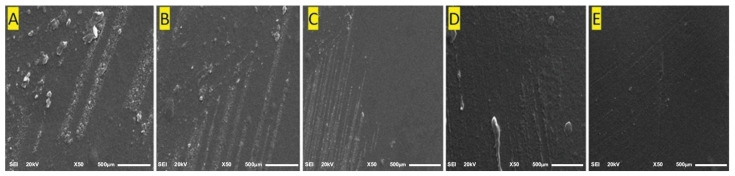
SEM images of hydrogel films: (**A**) batch F1 under 50×; (**B**) batch F2 under 50×; (**C**) batch F3 under 50×; (**D**) batch F4 under 50×; (**E**) batch F5 under 50×.

SEM images showing the surface methodology are presented in Figure 5A–E. The F5 batch formulated film presents a smooth surface with low protrusions (Figure 5E). The chitosan molecules are spread on PVA matrix, resulting in a mixture with good adhesive properties, while formulations containing lesser amounts of Chitosan have more abrasive surfaces, as exemplified by Figure 5A. Progressing from Figure 5A–E, for formulations F1 to F5 the surface becomes much smoother, as the curcumin particles are trapped inside the void spaces of the polymeric network.

### 2.8. Antimicrobial Study

**Figure 6:**
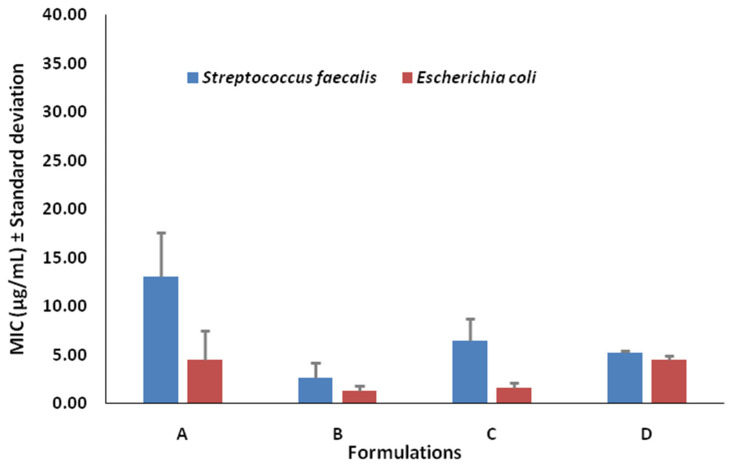
Antibacterial activity of formulated hydrogel F4 (in vitro MIC in µg/mL); the error bar represents the standard deviation of the mean (n = 3), where A is Curcumin, B is Chitosan + PVA + Curcumin, C is Chitosan + PVA and D is Chloramphenicol.

**Figure 7:**
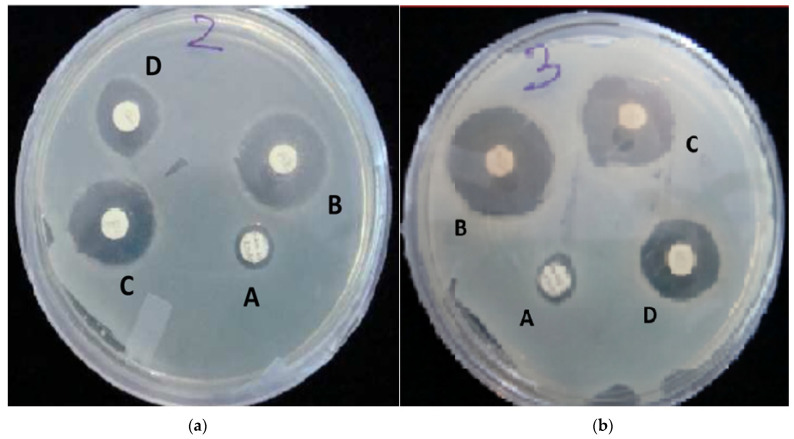
(**a**): Zone of inhibition of the formulated nanosheets against *Streptococcus faecalis* (Gram-positive) as evaluated by disc diffusion assay (A: Curcumin; B: Chitosan + PVA + Curcumin; C: Chitosan + PVA; D: Chloramphenicol). (**b**): Zone of inhibition of the formulated nanosheets against *Escherichia coli* (Gram-negative) as evaluated by disc diffusion assay (A: Curcumin; B: Chitosan + PVA + Curcumin; C: Chitosan + PVA; D: Chloramphenicol).

The antibacterial activity of films was assessed using the turbidity technique. Although the films were efficient against both gram-positive and gram-negative microorganisms, it was found that curcumin in pure form had a higher MIC (minimum inhibitory concentration) with respect to the bacteria (as measured in µg/mL) due to its incapacity to function alone. In addition, it was discovered that the film (F4 hydrogel) comprising chitosan, PVA, and curcumin had a low MIC, indicating that it had a higher inhibitory effect against gram-positive and gram-negative bacteria (Table 3 and Figure 6). The combination of chitosan and PVA has previously been shown to possess inhibitory action against bothgram-positive and gram-negative bacteria. The zones of inhibition of formulations for both types of bacteria are represented in Figure 7a,b.

### 2.9. In Vitro Drug Release

**Figure 8:**
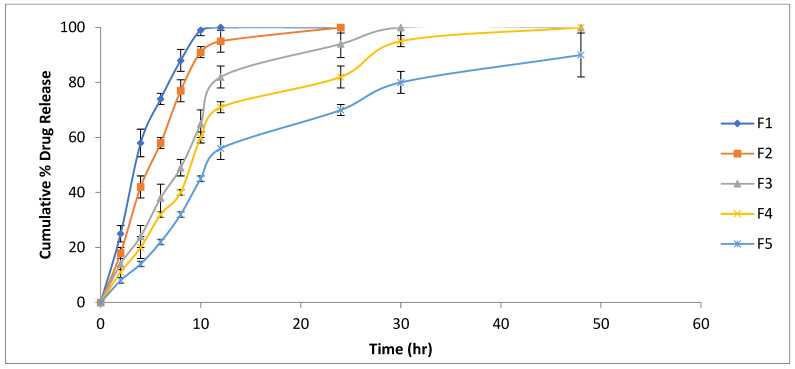
Drug release profiles of various batches of hydrogel (F1 to F5).

The in vitro drug release was studied for various batches of hydrogel films. The drug release test is indicative of release of curcumin for an extended period of time which governs the WH effect of films. It was found that as the concentration of chitosan increasesfrom F1 to F5, and a retardant release effect was observed. The molecular linkages between PVA and chitosan form a strong matrix, causing a decrease in the release of drug atoms. Many other researchers have reported similar effects for drug release based on polymer concentration [41]. After soaking in water, the hydrogel film instantly begins to swell. Water molecules permeate into the film’s structure during this phase. The polymer chains rearrange into more expanded conformations, and the free volumes within the hydrogel are enhanced as a result of the nature of PVA and chitosan and their favourable interactions with water [42]. When water enters the hydrogel structure, the curcumin molecules partly dissolve in the water and are easily able to diffuse via the holes formed by the swelling phenomena. The polymer chains partially dissolve when the hydrogel film is submerged in the releasing media. Because the hydrogel dissolves in the releasing media, curcumin may be released as well. Different drug release models, including the zero-order, first-order, Higuchi, Korsmeyer–Peppas, and Hixson–Crowell models, were adapted to the invitro drug release. The F1 formulation displayed remarkable results, as demonstrated in a prior study [40], whereas the remaining formulations were best suited to the Korsmeyer–Peppas model, as shown in Figure 8. The n-values of the F1–F3 formulations point to a Fickian-type diffusion during drug release, and the value of n was found to be anomalous based on the drug release pattern due to the presence of substantial amounts of polymer in formulations F4 and F5. This can be explained by the many releasing processes at work, including diffusion, erosion, and polymeric network relaxation.

### 2.10. Protein Targets and Ligand Preparations for Analysis 

In this study, targeted casein kinase-1 (CK1) (PDB ID: 3UZP) [43] and glycogen synthase kinase-3β (GSK3B) (PDB ID: 5HLP) [44] were used for molecular docking investigations. These two proteins are significant in improving the functions of the Wnt/β-catenin pathway. A recent study [45] has shown that the Wnt/β-catenin signalling pathway is activated when released Wnt proteins connect to a receptor complex made up of an FZD family member and LRP5/6 [46]. The Wnt/β-catenin pathway is made possible by a number of complicated proteins. Casein kinase-1 (CK1) and glycogen synthase kinase-3 (GSK3B) phosphorylate important parts of the Wnt/β-catenin signalling pathway, such as B-catenin, Axin, and APC (adenomatous polyposis coli), and act as negative or positive controllers of the pathway. Because of these phosphorylation events, Axin and APC stick together with b-catenin even more. CK1 is a group of serine/threonine-specific protein kinases that control different biological processes, such as Wnt signalling [47]. They act as both Wnt activators and Wnt inhibitors, as they phosphorylate several parts of the pathway. The CK1 family is made up of six human isoforms, which are found everywhere and share a highly similar kinase region. The N-terminal and C-terminal extensions of each protein are different. The amino-terminal kinase region is very similar in all of the members of the CK1 family [46,48]. The serine/threonine kinase GSK3B binds to and phosphorylates several proteins in the Wnt pathway, which makes β-catenin less active.

Rather than focusing on repair, which may lead to scarring, modern wound therapeutics instead attempt to promote a regenerative response that returns damaged skin to its pre-injury condition. As a result, the development of regenerative wound-healing therapies relies on our knowledge of the underlying molecular processes of the signaling pathways involved in regenerative healing. There are several steps involved in WH, and the Wnt/β-catenin pathway is crucial in many of them, including cell proliferation and tissue remodeling [49,50]. In addition, it has functions in the activation of stem cells and the production of growth factors. As a result, activating the Wnt/β-catenin pathway may be the best strategy for promoting wound regeneration. These proteins were prepared carefully for docking analysis; the respective ligands were removed and the same binding pockets reported in the literature were identified.

### 2.11. Molecular Docking

**Figure 9:**
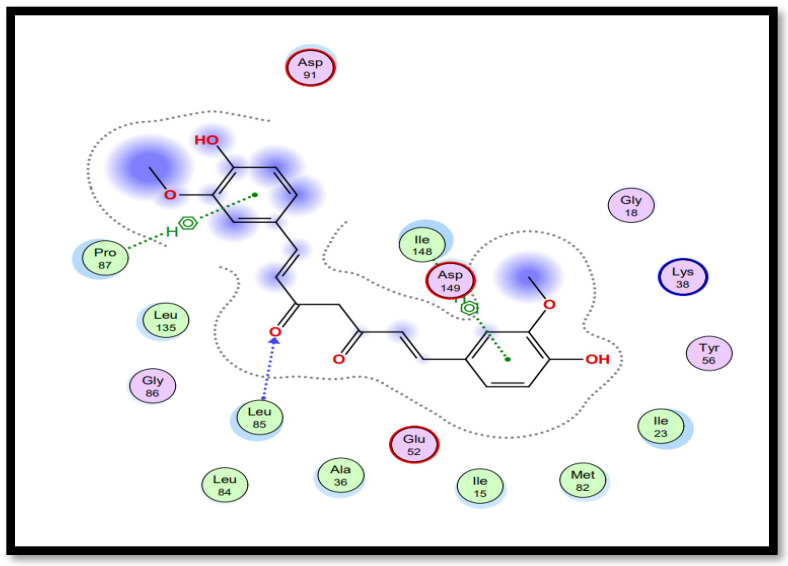
Interaction plot of docked ligand curcumin (CID_969516) within the active site of protein target casein kinase-1 (CK1) (PDB ID: 3UZP).

**Figure 10:**
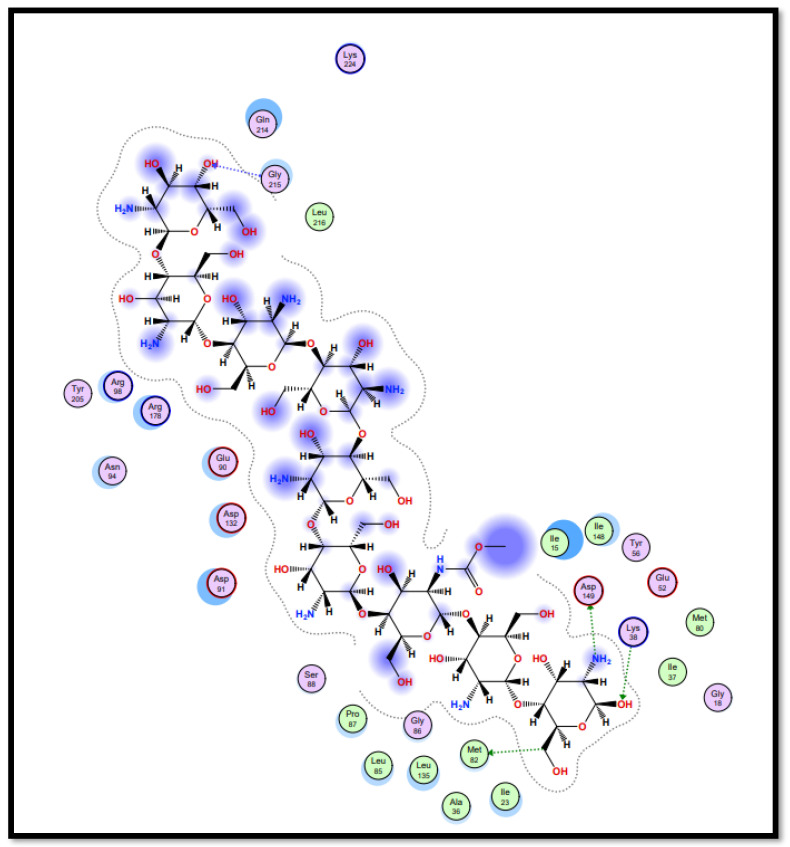
Interaction plot of docked ligand chitosan (CID_71853) within the active site of protein target casein kinase-1 (CK1) (PDB ID: 3UZP).

**Figure 11:**
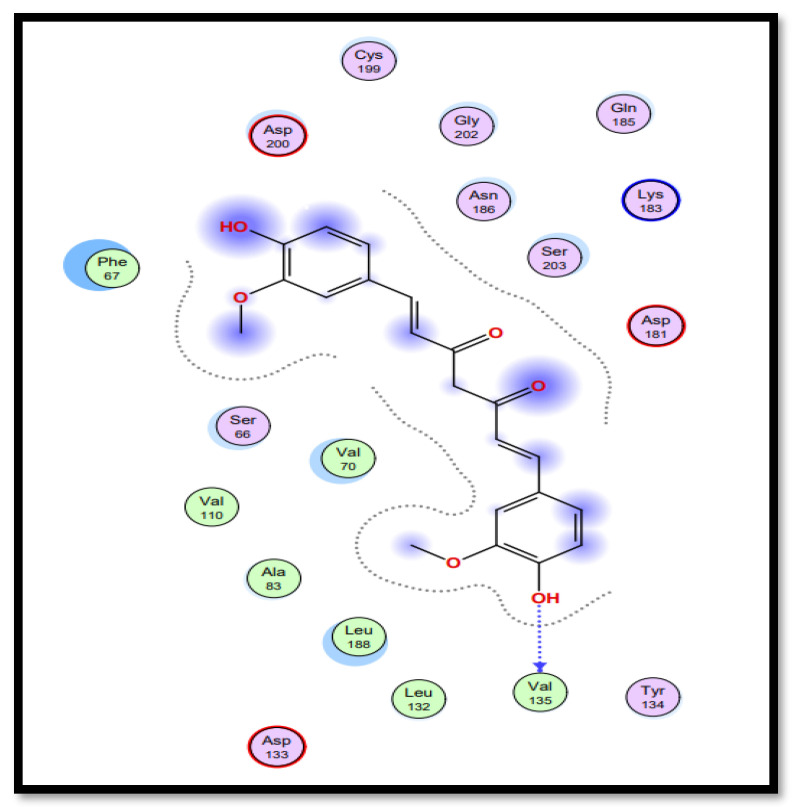
Interaction plot of docked ligand curcumin (CID_969516) within the active site of protein target glycogen synthase kinase-3β (GSK3B) (PDB ID: 5HLP).

**Figure 12:**
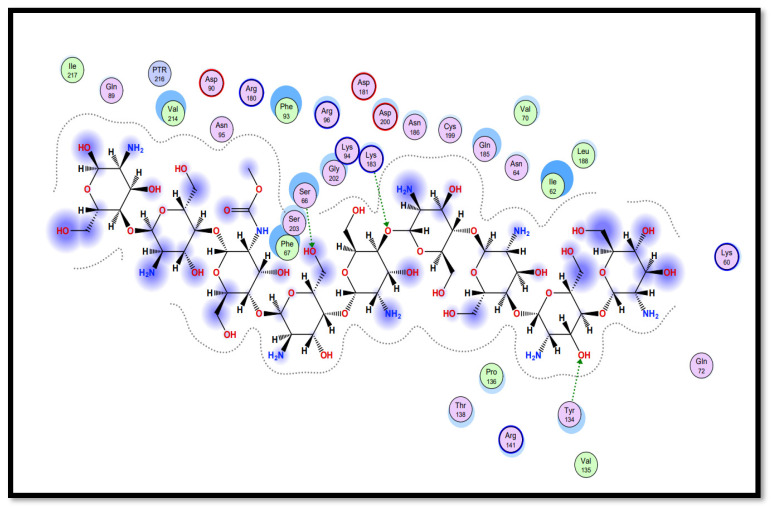
Interaction plot of docked ligand chitosan (CID_71853) within the active site of protein target glycogen synthase kinase-3β (GSK3B) (PDB ID: 5HLP).

Molecular docking studies of five identified components with two macromolecular proteins were performed using MOE software [51,52]. The binding free energies of these chemicals were obtained within optimal binding conformations, and it was observed that four ligands obtained good conformational results in terms of binding interaction and binding energies in kcal/mol, with the exception of polyvinyl alcohol; the results are shown in Table 4. The optimal binding conformations of the chemicals interacting in the region of the active site of both target proteins are demonstrated in Figure 9, Figure 10, Figure 11 and Figure 12.

Table 5 presents a summary of the interaction analysis of the two best virtual hits; curcumin (CID_969516) presents the best bounded conformation at −6.9875 kcal/mol, and presents three bonds with the LEU85 (A), PRO87 (A), and ILE 148 (A) residues of the CK1 target protein with bond distances of 3.22 Å, 4.01 Å, and 4.44 Å, respectively. The binding interactions of curcumin atoms and CK1 protein residues are presented in Figure 6. Chitosan (CID_71853) presents the best bounded conformation at −10.3979 kcl/mol, and presents four hydrogen bonds with the ASP149 (A), MET82 (A), GLY215 (A), and LYS38 (A) residues of the CK1 target protein, with respective bond distances of 3.18 Å, 3.90 Å, 3.17 Å, and 3.40 Å. The binding interactions of chitosan atoms with CK1 protein residues are presented in Figure 10.

Curcumin (CID_969516) presents the best bounded conformation at −6.5164 kcal/mol, and presents one hydrogen bond with the VAL135 (A) residue of the GSK3B target protein, with a bond distance of 3.16 Å. The binding interactions of curcumin atoms with GSK3B protein residues are presented in Figure 11. Chitosan (CID_71853) presents the best bounded conformation at −10.5200 kcal/mol, and presents four hydrogen bonds with LYS183 (A), TYR134 (A), and SER66 (A) residues of the GSK3B target protein, with bond distances of 3.55 Å, 3.00 Å, and 2.93 Å, respectively. The binding interactions of chitosan atoms with GSK3B protein residues are presented in Figure 12.

## 3. Conclusions

Chitosan–PVA hydrogel films loaded with curcumin were formulated and assessed for their invitro and insilico characteristics with respect to their WH potential. The physical tests, including WVTR, swelling ratio, invitro drug release, and mechanical strength are in good agreement with the characteristics required for WH potential. An additional antimicrobial study was carried out for both gram-positive and gram-negative bacteria, and it was found that the MIC for hydrogel film formulation was quite low in comparison to both drug and placebo. These results show that the hydrogel film contained significant antimicrobial activity even at low amounts of drug. In addition, insilico studies were conducted to demonstrate the WH activity. Further invitro studies are required for evaluation of WH potential. In summary, the outcomes of our in vitro and in silico investigations offer encouraging new perspectives on this research problem. However, we are aware of the significance of confirming these results in a live environment. For the purpose of bolstering and supporting our findings, we are dedicated to carrying out extensive and meticulous in vivo investigations. A better comprehension of the processes behind our study topic can be attained by integrating in vitro, in silico, and in vivo experimental data, which is expected to further progress the discipline.

## 4. Materials and Methods

### 4.1. Materials

Curcumin (Product Code: C1386-5G) and Chitosan (Product Code: 448869-50G) were purchased from Sigma Aldrich, Saint Louis, USA, while PVA was bought from Loba Chemicals Pvt. Ltd. Mumbai India. All other reagents and chemicals were of analytical grade. The hydrogel films were prepared using physical crosslinking via the solvent casting method. Chitosan solutions with varying concentrations were prepared by dissolving a weighed amount of chitosan in acetic acid solution (3% *v*/*v*) with constant stirring for about 2 h. In parallel, PVA solution (5% *w*/*v*) was prepared by dissolving a weighed quantity in distilled water with constant stirring at 50 °C for 4 h. Varying ratios of chitosan and PVA were mixed together, as shown in Table 6, and curcumin (100 mg) was added to each diluted mixture via mechanical blending at 1000 rpm for 10 min. The solution was then transferred into Pyrex petri plates and allowed to air dry at room temperature for 48 h. Dried films were peeled from the petri plates and stored in a desiccator for further evaluation. The whole process was carried out using low-actinic glassware, and Sodium D light was used during the drying process. The formulation concentrations are shown in Table 6.

### 4.2. Methods

#### 4.2.1. Thickness and Weight Variation

The thickness of the prepared hydrogel films was checked using a calibrated digital vernier caliper (Make Mitutoyo, Takatsu-ku, Japan). For the weigh measurement, the films were weighed individually. All analyses were performed in triplicate, and the standard deviations were calculated based on observation.

#### 4.2.2. Folding Endurance

Folding endurance provides an estimate of how many times a film can be folded on single plane without breaking or forming visible cracks. The experiment was conducted in triplicate.

#### 4.2.3. Moisture Content

It is necessary to evaluate the moisture level in hydrogels, as they naturally contain water. The films were initially weighed for estimation purposes, then dehydrated for around 24 h using silica gel. The films were weighed several times until a steady weight was noted.

The moisture content was determined as per the following equation:

Moisture Content (%) = (W_i_ − W_d_)/W_d_ × 100.

The moisture content determination experiment was performed in triplicate.

#### 4.2.4. Moisture Uptake

The films were weighed initially and placed in desiccatorcontaining silica gel (activated) for 24 h.The films were then collected and transferred to another desiccator containing saturated sodium chloride solution for another 72 h, with relative humidity maintained at 75%. The final weight of the films was recorded, and their moisture uptake capacity was determined as per following equation:

Moisture uptake (%) = (W_m_ − W_i_)/W_i_ × 100.

The moisture uptake experiment was performed in triplicate.

#### 4.2.5. Swelling Ratio

The dried hydrogel sheets were sliced into square specimens measuring 2 × 2 cm^2^. The samples were measured out and stored in 250 mL of phosphate buffer at a temperature of 25 °C. The samples were weighed after being blotted with tissue paper to remove extra surface water at regular intervals. The evaluation was performed three times. The swelling ratio was calculated using the following formula:

Swelling ratio (%) = (W_s_ − W_d_)/W_d_ × 100.

In the above formula, W_d_ is the initial weight of the dry film samples and W_s_ is the weight of swollen film samples. The experiment was performed in triplicate.

#### 4.2.6. WVTR

The WVTR test was performed with reference to ASTM D6701-21 [53]. The samples were mounted in the mouth of a polytop glass containing about 10 mL of phosphate buffer with a pH of 7.4. The samples were pre-weighed and heated in an oven for 24 h at 35 °C. WVTR was determined using the following formula:

WVTR = W_i_ − W_t_/A × 106 g/m^2^day^−1^

where A is the polytop opening area (mm^2^) and W_i_ and W_t_ are the polytop weight before and after being placed in the oven, respectively. The experiment was performed in triplicate.

#### 4.2.7. Mechanical Properties

A certain amount of mechanical resistance to stretching or distortion was expected from the films. Therefore, the tensile strength (N/mm^2^) and percent elongation at break of the material were assessed using a texture analyzer (TA XT plus, Stable Microsystem, Godalming, Surrey, UK) and a 5 kg loaded cell. The films were sampled using a 1 cm^2^ sample area clamped with a 50 mm/min separation rate, and were assessed in triplicate. 

#### 4.2.8. FTIR

For the evaluation of different interactions taking place during interaction of chitosan, PVA, and curcumin, the films were evaluated using the FTIR technique. The films were dried in vacuum and evaluated using a Perkin-Elmer spectrometer (Spectrum two, Model no.L160000A, Waltham, MA, USA) with a scanning range of 4000–650 cm^−1^ and a resolution of 4 cm^−1^. 

#### 4.2.9. SEM

Morphological evaluation of hydrogel films was performed using a scanning electron microscope (S 4300 SE/N, Hitachi, Tokyo, Japan) with an accelerating voltage of 15 kV. All the samples were staged on the metallic stub, adhered using double-sided tape, and coated with a layer of gold. 

#### 4.2.10. Antimicrobial Activity

The antibacterial activities of all the samples were studied against *Streptococcus faecalis* and *E. coli*, which were used as test organisms to determine the antibacterial effects of all the substances. The CLSI recommendations for the broth microdilution technique were used to calculate the MIC values [54]. In brief, bacterial strains were cultivated on Luria Bertani Agar medium, which has a pH of 7.5 ± 0.2 and contains 0.5% peptone, 0.5% yeast extract, 1% NaCl, and 1.5% agar. Then, 10 mg of hydrogel was suspended in 10 mL of PBS (phosphate buffer 67 mM, pH 7.4, 0.05% Tween 20, and 0.02% sodium azide) in borosilicate vials to create the stock solution. The gel particle solutions were incubated for 24 h at 37 °C while being shaken; 500 µL of the supernatant was centrifuged and kept at 4 °C until the antibacterial activity could be examined. The positive control was the common antibiotic chloramphenicol, while the negative control was PBS. Sterilized Mueller–Hinton Broth (MHB), which contains 30% beef extract, 1.75 percent casein acid hydrolysate, and 0.15% starch, and has a pH of 7.4 ± 0.2, was then supplemented with the bacterial cultures. The MIC was calculated using two-fold serial dilution in MHB medium containing 1.95–1000 µg/mL of the test compounds; 150 µL of medium (MHB) was collected in duplicate and applied to each well of a 96-well microtitre plate, along with 10 µL of 0.5 McFarland standard (1.5 × 10^8^ CFU/mL) culture pathogens from MHB. The inoculation plates were incubated at 37 °C For 24 h.

#### 4.2.11. In Vitro Drug Release

Fixed-size hydrogel sheets were placed on a glass slide and fastened to a stainless steel mesh. The samples were analyzed using a Paddle-type Lab India DS8000 (New Delhi, India) at a temperature of 37 ± 0.5 °C and 50 rpm while being submerged in phosphate buffer pH 7.5 as the dissolving medium. At regular intervals, aliquot samples were taken and the amount of curcumin emitted was measured using a UV/visible spectrophotometer for spectrophotometric analysis at 424 nm (2202, Systronics, Ahmedabad, India). To assess the mechanism of drug release from the hydrogel films, the in vitro data were fitted to a variety of kinetic models, including the zero-order, first-order, Higuchi, Hixson–Crowell, and Korsmeyer–Peppas models.

Zero-order: Q = Q_o_ + k_ot_

First-order: lnQ = lnQ_o_ + k_1t_

Higuchi model: Q = k_Ht12/_

Hixson–Crowell model: Q_o1/3_ − Q_R1/3_ = K_st_

Korsmeyer–Peppas model: QQ_t/_ = Kk_ptn_

where Q is the amount of drug released at time t, Q_o_ is the initial amount of drug, Q_R_ is the amount of drug remaining at time t, and Q_t_ is the total amount of drug release; k_o_, k_1_, k_H_, and k_KP_ are the kinetic constants for the zero-order, first-order, Higuchi, Hixson–Crowell and Korsmeyer–Peppas models, respectively, and n is the release exponent. The in vitro dissolution experiment was performed in triplicate.

#### 4.2.12. Selection of Protein Targets and Chemical Compounds

**Figure 13:**
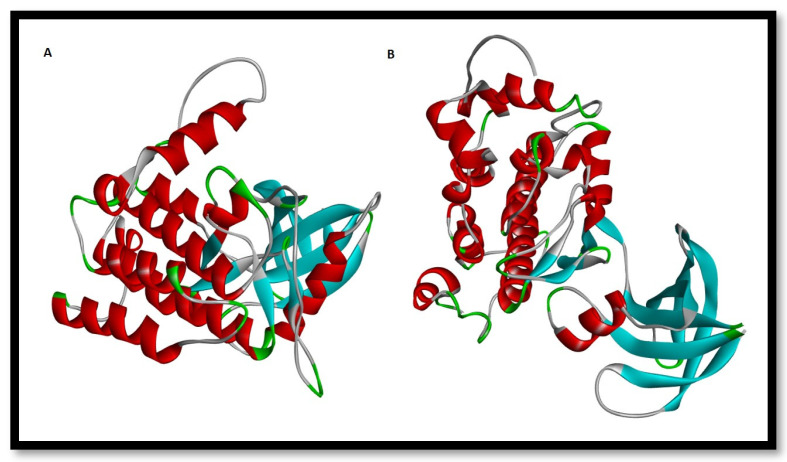
Three-dimensional representation of (**A**) Protein target casein kinase-1 (CK1) (PDB ID: 3UZP) and (**B**) Protein target glycogen synthase kinase-3β (GSK3B) (PDB ID: 5HLP).

In pharmaceutical research and computer-aided drug design (CADD), one of the most significant computational techniques is called molecular docking [55,56]. The fundamental purpose of molecular docking is to find probable binding geometries of a suspected ligand with an acknowledged/identified structure of a target protein [57]. This can be accomplished by comparing the three-dimensional structures of both the hypothetical ligand and the targeting protein. The chemical structures of the selected compounds were drawn using the ChemDraw tool for further analysis [58,59]. In this study, we targeted casein kinase-1 (CK1) (PDB ID: 3UZP) [43] and glycogen synthase kinase-3β (GSK3B) (PDB ID: 5HLP) (Figure 13A,B) [44], as they are involved in the Wnt/β-catenin signaling pathway.

#### 4.2.13. Molecular Docking

Information on the macromolecular mechanism of a bound ligand near the active site of a target protein may be assisted using molecular docking, an insilico application that provides an explanatory scoring scheme and best binding poses generated by a best-docked complex that aids in retrieval of protein–ligand binding interaction [60,61].Five ligands were found to interact with the target proteins (PDB IDs: 3UZP and 5HLP) via molecular docking. The PDB format was imported to the molecular operating environment (MOE) software [51,52]. To prepare proteins for the docking technique, we eliminated heteroatoms, 3D protonation, water molecules, and the default ligand linked to the target protein. Known active sites were identified in each protein structure [43,44], and structural optimization was achieved by subsequent constraints such as the addition of hydrogen atoms. Energy minimization with the Amber14 force field method was performed with both chiral and geometrical constraints. The MOE program generates a database of five compounds selected from experimental research for molecular docking simulations, which is then stored as an MDB file for further examination. Refinement and computation of the binding free energies (G), assessed using the scoring function, were applied to the top-ranked postures (GBVI/WSA dg) [62]. A reliable scoring scheme resulting in the docking score of the correct binding poses was established based on the number of molecular interactions (hydrogen, Pi, and hydrophobic interactions) [52,55,56,57,59,60,61,62,63,64]. The MOE database of the docked complex was visualized carefully in order to understand the mode of binding interactions of the ligands bound in the selected pocket of the target protein.

## Figures and Tables

**Table 1:** Physicochemical evaluation of hydrogel films (n = 3).

Formulation Code	Thickness (mm)	Weight Variation (g)	Folding Endurance	Moisture Content (%)	Moisture Uptake (%)
F1	0.039 ± 0.004	0.417 ± 0.03	345 ± 12	17.14 ± 1.12	10.28 ± 0.04
F2	0.041 ± 0.005	0.421 ± 0.05	414 ± 14	19.14 ± 2.25	12.14 ± 1.14
F3	0.049 ± 0.008	0.454 ± 0.04	434 ± 10	21.24 ± 1.24	13.24 ± 0.15
F4	0.054 ± 0.007	0.472 ± 0.03	433 ± 12	22.74 ± 1.14	13.75 ± 0.14
F5	0.057 ± 0.006	0.495 ± 0.02	478 ± 14	26.72 ± 2.17	15.47 ± 0.08

**Table 2:** Tensile strength, elongation at break, and WVTR parameters for the produced batches of hydrogel films (n = 3).

Formulation Code	WVTR (g/m^2^/Day)	Tensile Strength (N)	Elongation at Break (mm)
F1	2710.54 ± 15.96	4.14 ± 0.24.	30.14 ± 1.49
F2	2541.12 ± 58.47	4.74 ± 0.83	32.48 ± 5.14
F3	2198.65 ± 65.25	21.52 ± 2.84	33.65 ± 4.58
F4	1895.62 ± 54.51	28.65 ± 2.98	34.21 ± 2.98
F5	1630.70 ± 25.68	38.87 ± 5.24	35.78 ± 2.58

**Table 3:** Antibacterial activity of formulated hydrogels (in vitro MIC in µg/mL).

Sr No.	Formulations	MIC (µg/mL) ± Standard Deviation	
		*Streptococcus faecalis* (Gram Positive)	*Escherichia coli* (Gram Negative)
1	Curcumin (A)	13.02 ± 4.51	4.55 ± 2.98
2	Chitosan + PVA + Curcumin (B)	2.60 ± 1.13	1.30 ± 0.56
3	Chitosan + PVA (C)	6.51 ± 2.26	1.62 ± 0.57
4	Chloramphenicol (D)	5.25 ± 0.21	4.51 ± 0.46

**Table 4:** Selected ligand and target protein molecular docking results.

Ligand Names	PubChem ID	Ligand Structures	Selected Target Protein	
			CSK- Binding Energies (Kcal/mol)	GSK3B- Binding Energies (Kcal/mol)
Polyvinyl alcohol	11199	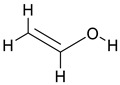	−3.0805	−2.6834
Curcumin	969516	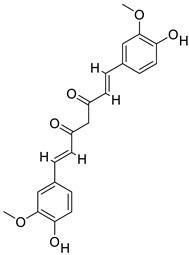	−6.9875	−6.5164
Curcumin III	5315472	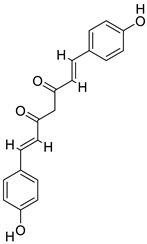	−6.0156	−5.9395
Curcumin II	5469424	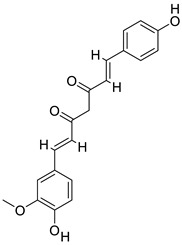	−6.6456	−5.9991
Chitosan	71853	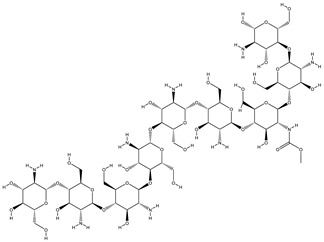	−10.3979	−10.5200

**Table 5:** Summary of interaction analysis for the two best virtual hits.

Ligand Name	Binding Energy (Kcal/mol)	Binding Interaction			
		Interacting Residues	Interaction Type	Bond Distance	Bond Energy (Kcal/mol)
Protein target casein kinase-1 (CK1) [PDB ID: 3UZP]					
Curcumin (CID_969516)	−6.9875	O5–N LEU85 (A) 6-ring CA PRO87 (A) 6-ring CD1 ILE 148(A)	H-acceptor Pi-H Pi-H	3.22 4.01 4.44	−0.9 −0.6 −0.5
Chitosan (CID_71853)	−10.3979	N48–OD2 ASP149 (A) C102–SD MET82 (A) O34–N GLY215 (A) O35–NZ LYS38 (A)	H-donor H-donor H-acceptor H-acceptor	3.18 3.90 3.17 3.40	−0.5 −0.5 −1.2 −0.6
Protein target glycogen synthase kinase-3β (GSK3B) [PDB ID: 5HLP]					
Curcumin (CID_969516)	−6.5164	O3–OVAL135 (A)	H-donor	3.16	−1.0
Chitosan (CID_71853)	−10.5200	O3–NZ LYS183 (A) O22–OH TYR134 (A) O25–OG SER66 (A)	H-acceptor H-acceptor H-acceptor	3.55 3.00 2.93	−0.6 −1.4 −1.4

**Table 6:** Composition table used for preparing different batches of hydrogel films.

Formulation Code	Chitosan Solution		PVA Solution (5% *w*/*v*) (mL)	Curcumin (mg)
	Concentration of Chitosan (% *w*/*v*)	Amount of Chitosan (mL)		
F1	0.25	80	20	100
F2	0.50	80	20	100
F3	0.75	80	20	100
F4	1.0	80	20	100
F5	2.0	80	20	100

## Data Availability

Data can be accessed upon request to the corresponding author.
